# 

**Published:** 1921-09-10

**Authors:** 


September 10, 1921] [Price Twopence

### The Hospital

The Workers' Newspaper of Administrative Medicine and Institutional Life, Administration, National Insurance and Health.

### CONTENTS

**Hospital Treatment as an Additional Benefit** ... 393
**The Prohibition Issue** ... 394
**Modified Optimism** ... 394
**Notes of the Week** ... 395
Prince Henry and Hexham—The Prince as Landlord—Barrister, Surgeon, Magistrate—Hospital Tennis—Infant Mortality in Summer—Venereal Disease—The London "Toll" Exchange—Epilepsy—Massage by the Blind—The Surplus of the Approved Societies—Economy in Whitehall—The Syllabus of the General Nursing Council—Hospital Staffs and Unemployment Insurance—"The Mission Hospital"—Warrington's New Sanatorium.
**The Fracture Department** ... 397
A New Departure and a Valuable Analysis.
**Health Problems in India** ... 398
**The Problem of the Insane** ... 399
IV.—Asylum Medical Work.
**The London County Council Health Report** ... 400
Statistics and Conclusions.
**The Public Well-Being** ... 401
The Census Report.
**The Editor's Letter-Box** ... 402
"Higher Charges for Distant Patients"—The Charity Organisation Society—"Insanity Can be Cured."
**The Matrons' and Sisters' Department** ... 403
The Bonus Decrease.
**Round the Hospitals** ... 403
**Trained Nurses for Child-Welfare Schemes** ... 405
**Schedule of Maternity Home Fees** ... 405
**A Cheerful Report from Ipswich** ... 406
**Opening of Grosvenor Telephone Exchange** ... 406
**A Maximum of Seventy Beds per Doctor** ... 406
**The College of Nursing (Limited by Guarantee)** v
**General Nursing Council for Scotland** ... v
**Matrons' and Sisters' Appointments** ... v

